# Influence of Fluorinated Substituents on the Near-Infrared Phosphorescence of 5d Metallocorroles

**DOI:** 10.1021/acsorginorgau.3c00016

**Published:** 2023-07-12

**Authors:** Krister Engedal Johannessen, Martin Amund Langaas Johansen, Rune F. Einrem, Laura J. McCormick McPherson, Abraham B. Alemayehu, Sergey M. Borisov, Abhik Ghosh

**Affiliations:** † Department of Chemistry, UiT−The Arctic University of Norway, 9037 Tromsø, Norway; ‡ EPSRC National Crystallography Service, School of Chemistry, University of Southampton, Highfield, Southampton SO17 1BJ, U.K.; § Institute of Analytical Chemistry and Food Chemistry, Graz University of Technology, Stremayrgasse 9, 8010 Graz, Austria

**Keywords:** Fluorinated compounds, fluorous, corrole, phosphorescence, triplet photosensitizer, gold, rhenium

## Abstract

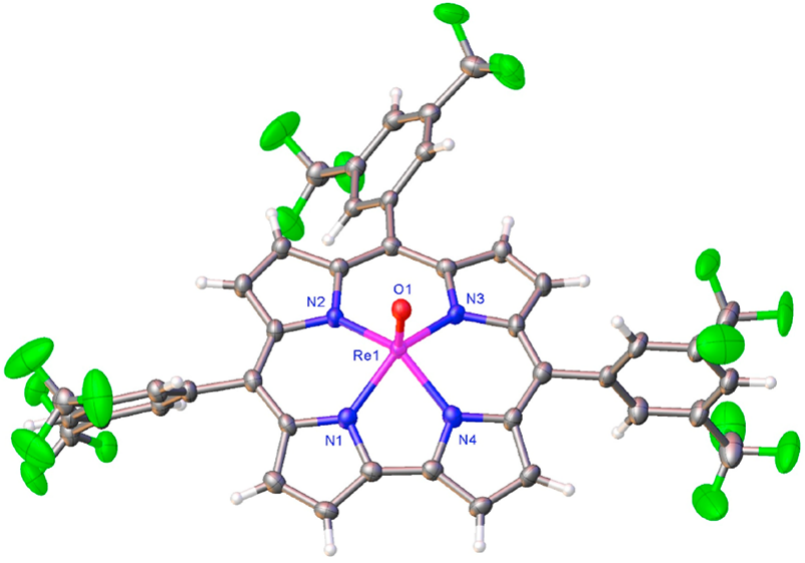

The influence of fluorinated substituents on the luminescent properties of rhenium-oxo, osmium-nitrido, and gold triarylcorroles was studied via a comparison of four ligands: triphenylcorrole (TPC), tris(*p*-trifluoromethylphenyl)corrole (T*p*CF_3_PC), tris{3,5-bis(trifluoromethyl)phenyl}corrole (T3,5-CF_3_PC), and tris(pentafluorophenyl)corrole (TPFPC). For each metal series examined, fluorinated substituents were found to enhance the luminescent properties, with the phosphorescence quantum yields and triplet decay times increasing in the order TPC < T*p*CF_3_PC < T3,5-CF_3_PC < TPFPC. Among the 11 complexes examined, the highest phosphorescence quantum yield, 2.2%, was recorded for Re[TPFPC](O).

The last decade has witnessed the emergence of a unique class of transition metal complexes, the 5d metallocorroles.^1^ Their uniqueness derives from their size-mismatched nature, which involves a large 5d ion encapsulated by a sterically constrained, macrocyclic corrole ligand.^2−4^ In spite of the steric strain inherent in their structures, the middle and late 5d transition metal (Re,^5−8^ Os,^9^ Ir,^10^ Pt,^11,12^ and Au^13−20^) corroles have proved thermally and photochemically rugged. Furthermore, their photophysical properties are conducive to applications as photosensitizers, most notably in photodynamic therapy and oxygen sensing.^21−33^ Interestingly, in the course of our photophysical studies on 5d metallotriarylcorroles, we observed somewhat higher phosphorescence quantum yields for tris{(*p*-trifluoromethyl)phenyl}corrole complexes than for their more electron-rich counterparts.^24,28−30^ The observation made us wonder whether fluorinated substituents might have a beneficial effect on the luminescence properties of 5d metallocorroles. A photophysical study was accordingly carried out on the complexes depicted in Chart 1, except for the M = OsN, Ar = C_6_F_5_ case, which was not studied because of synthetic difficulties. We found that fluorinated substituents indeed appear to have a beneficial effect on the luminescence properties of the complexes, significantly increasing both the phosphorescence quantum yields and the triplet decay times.

**Chart 1:**
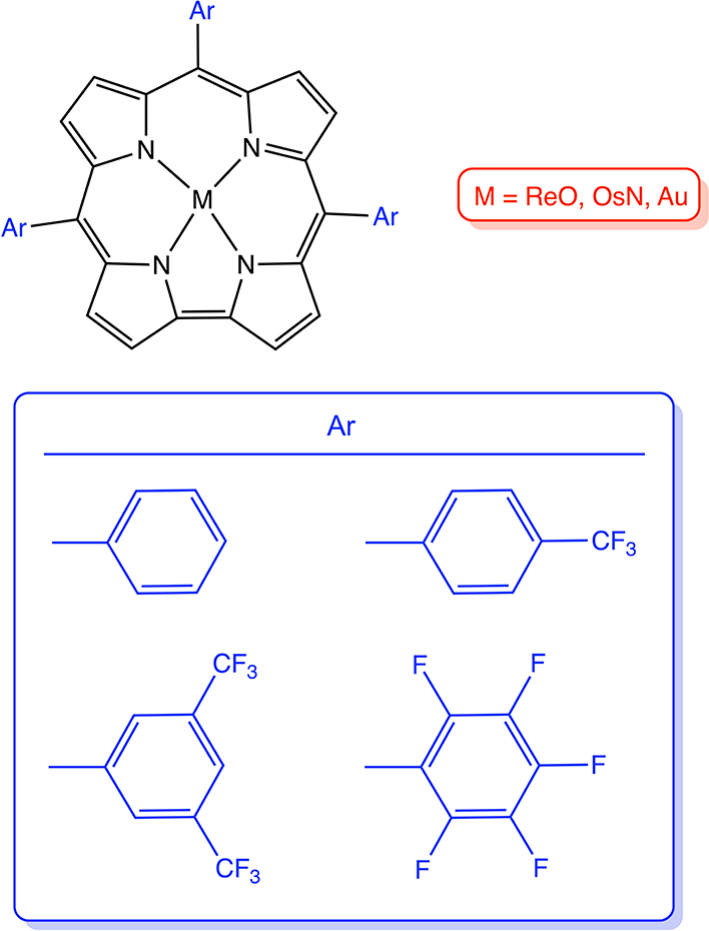
Molecules Studied in This Work

The influence of fluorinated substituents on the luminescence properties of rhenium-oxo, osmium-nitrido, and gold triarylcorroles was studied via a comparison of four ligands: triphenylcorrole (TPC), tris(*p*-trifluoromethylphenyl)corrole (T*p*CF_3_PC), tris{3,5-bis(trifluoromethyl)}corrole (T3,5-CF_3_PC), and tris(pentafluorophenyl)corrole (TPFPC). The majority of the compounds in question have been previously synthesized;^5,9,15^ four new compounds were synthesized specifically for this study, namely, Re[T3,5-CF_3_PC](O), Os[T3,5-CF_3_PC](N), Au[T3,5-CF_3_PC], and Re[TPFPC](O). Unfortunately, Os[TPFPC](N) could not be synthesized because the azide used as part of the synthetic protocol resulted in nucleophilic displacement of the *para*-fluorines in the TPFPC ligand (consonant with multiple similar reactions in the literature^34−36^). Aside from that, the syntheses of the new compounds proved uneventful, and one, Re[T3,5-CF_3_PC](O), yielded a single-crystal X-ray structure (Figure 1 and Table S1). Key photophysical and electrochemical properties of the compounds are listed in Table 1.

**Figure 1:**
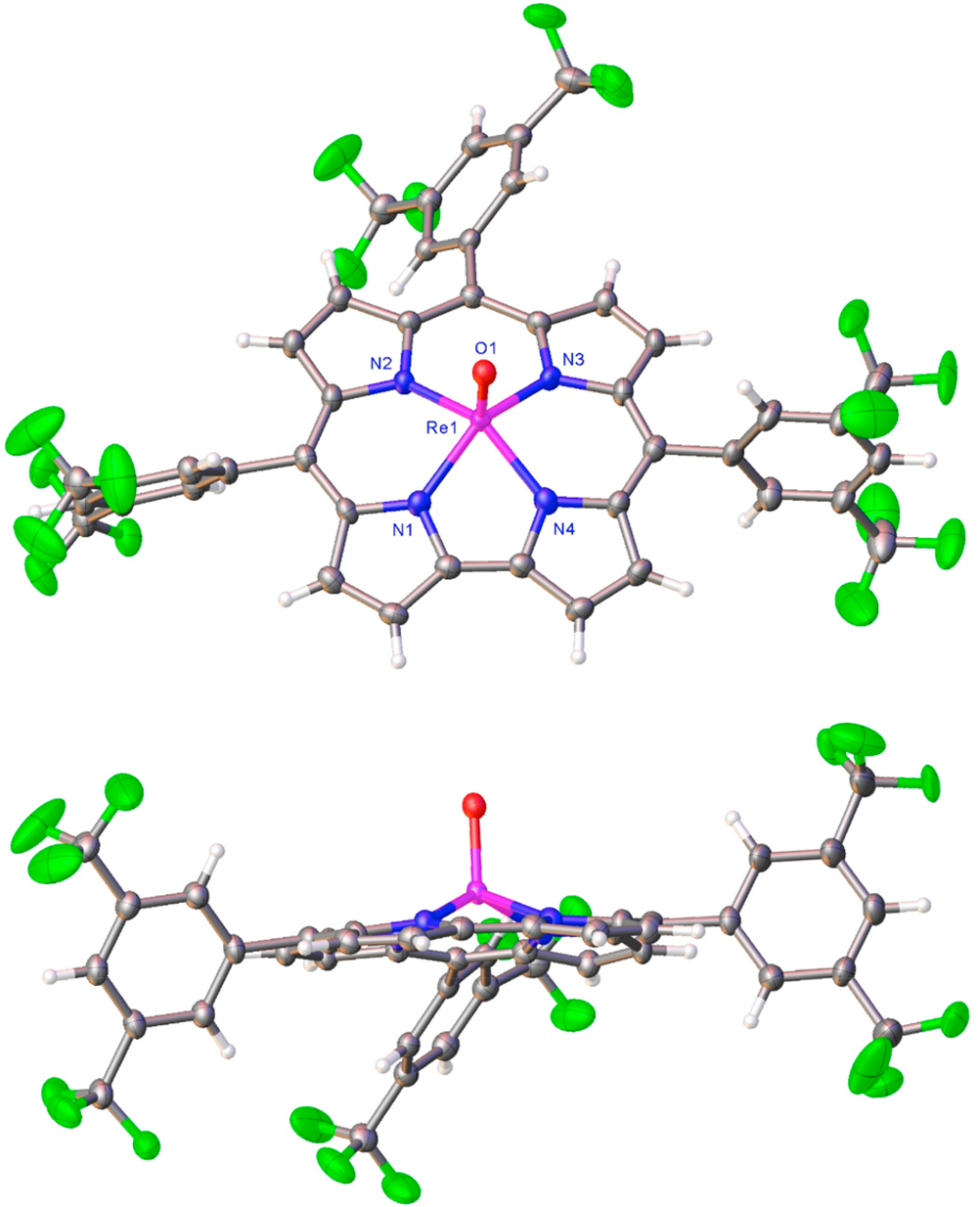
Two views of the thermal ellipsoid plot for Re[T3,5-CF_3_PC](O) at 50% probability. Selected distances (Å): Re1–N1 1.992(3), Re1–N2 2.006(3), Re1–N3 2.015(3), Re1–N4 1.996(3), and Re1–O1 1.574(3) Å.

**Table 1:** Photophysical and Electrochemical Properties of ReO, OsN, and Au Triarylcorroles in Anoxic Toluene (23 °C)

complex	λ_max_ abs, nm	λ_max-phos_ (nm)	Φ_phos_ (%)	τ_phos_ (μs)	*E*_1/2,ox1_ (V)	*E*_1/2,red1_ (V)	*E*_1/2,red2_ (V)	ref
Re[TPC](O)	440, 554, 586	776 (770)^a^	1.2	60	0.98	–1.26		(5), (29)
Re[T*p*CF_3_PC](O)	440, 553, 586	768 (777)^a^	1.4 (1.5)^a^	74	1.10	–1.16		(5), (29)
Re[T3,5-CF_3_PC](O)	440, 553, 586	760	1.6	75	1.21	–1.11	–1.64	this work
Re[TPFPC](O)	437, 552, 586	753	2.2	99	1.31	–1.04	–1.68	this work
Os[TPC](N)	444, 554, 595	784	0.64 (0.54)^b^	125 (128)^c^	0.91	–1.28		(9), (24)
Os[T*p*CF_3_PC](N)	444, 554, 593	778	0.7 (0.54)^b^	139 (150)^c^	1.02	–1.19		(9), (24)
Os[T3,5-CF_3_PC](N)	443, 553, 588	770	0.81	155	1.12	–1.10	–1.62	this work
Au[TPC]	421, 494, 532, 561, 575	792	0.23 (0.18)^d^	94 (86)^d^	0.80	–1.38		(15)
Au[T*p*CF_3_PC]	423, 494, 532, 562 (sh), 575	786	0.26 (0.19)^d^	97 (98)^d^	0.94	–1.29		(15)
Au[T3,5-CF_3_PC]	421, 493, 529, 567	777	0.33	99	1.05	–1.19	–1.62	this work
Au[TPFPC]	415, 491, 527, 561	751	0.68	170	1.18	–1.11	–1.68	this work

a Ref (29); excitation in the Q-band.

b Ref (24); the Φ_phos_ values have been recalculated based on the corrected value (21%) for the standard platinum(II) tetraphenyltetrabenzoporphyrin (Pt[TPTBP]).^37^

c Ref (24); frequency domain measurement.

d Ref (23).

All of the complexes proved emissive in deoxygenated toluene at room temperature (Figure 2 and Table 1). The emission was efficiently quenched by molecular oxygen and is thus ascribed to phosphorescence. The absorption and excitation spectra (Figures S19–S23) proved virtually identical, indicating that the emission originates solely from the metal complexes while also confirming the purity of the compounds. Although the emission spectra of the T3,5-CF_3_PC and TPFPC complexes are generally similar to those of the previously studied TPC and T*p*CF_3_PC complexes (which were also remeasured in this study), the emission maxima were found to shift hypsochromically with increasing electron-withdrawing character of the *meso*-aryl substituents; this effect was observed for all three metal series examined.

**Figure 2:**
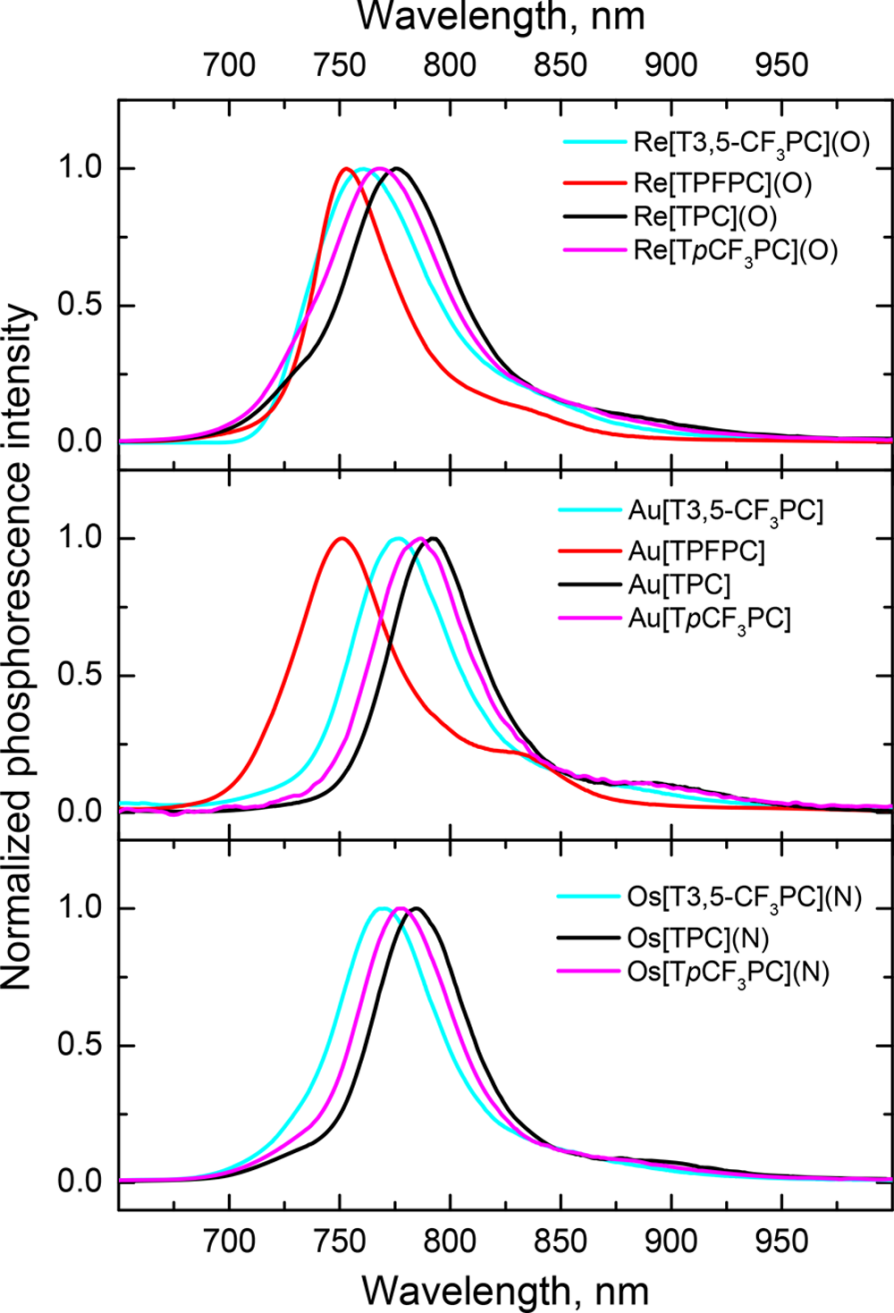
Emission spectra of the complexes in anoxic toluene at 23 °C. Excitation into the maximum of the Soret band of the complexes was performed.

As shown in Table 1, fluorination results in an increase in both luminescence quantum yields and decay times in the order TPC < T*p*CF_3_PC < T3,5-CF_3_PC < TPFPC, which is also the order of the redox potentials (see Figures S9–S13 for selected cyclic voltammograms). Figure 3 presents a graphical representation of the quantum yields for the different complexes. The ReO complexes are by far the strongest emitters, followed by the OsN, and last by the Au (Figure 3, upper panel). Notably, compared with their TPC analogues, the luminescence of Au[TPFPC] is enhanced much more strongly than that of Re[TPFPC](O) (Figure 3, lower panel). Thus, whereas the phosphorescence quantum yield triples on going from Au[TPC] to Au[TPFPC], the enhancement is less than double for their ReO counterparts. As a result of the fluorination-mediated enhancement, Au[TPFPC] emits as efficiently as Os[TPC](N). The trend in the luminescence decay times parallels that observed for the luminescence quantum yields (Table 1). The decay time of Au[TPFPC] is thus much longer (170 μs) than that of the other Au triarylcorroles (94–99 μs). Interestingly, although the parallelism is far from exact, the present findings appear similar to those of Liu and co-workers, who observed fluorination-induced enhancements of triplet quantum yields for free-base and gallium triarylcorroles.^38^

**Figure 3:**
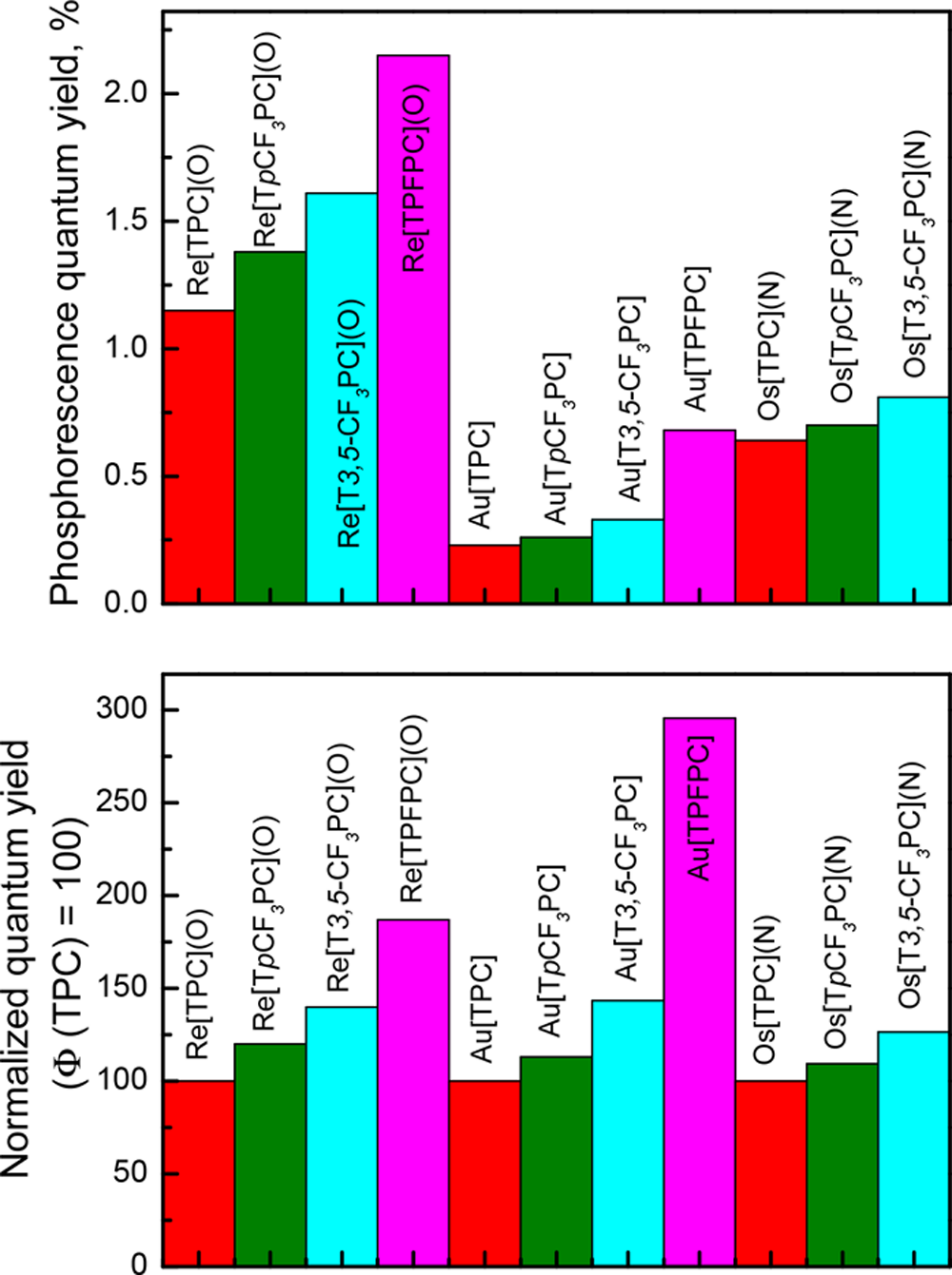
Phosphorescence quantum yields of the ReO, OsN, and Au corroles. The lower plot depicts the enhancement of the quantum yield upon fluorination: the values are normalized for the quantum yields of the TPC complex of each metal; i.e., the *Φ*_phos_ values of Re[TPC](O), Os[TPC](N), and Au[TPC] are each set as 100%.

The fact that the order of phosphorescence quantum yields parallels the order of redox potentials for each of three series of 5d metallocorroles suggests that the mechanism of enhanced luminescence is largely electronic in origin. However, the *ortho* fluorines in the TPFPC complexes may confer some degree of conformational rigidity, leading to increased triplet lifetimes. Fluorination also has a major impact on solute–solvent interactions, which, in turn, may also affect the luminescence properties. At this point, these potential influences remain to be disentangled, and the striking impact of fluorination is simply presented as an empirical observation.

In conclusion, introduction of fluorinated substituents onto the *meso*-phenyl groups results in enhancement of the luminescence properties of all three series of 5d metallocorroles: ReO, OsN, and Au. Substitution of phenyl groups by pentafluorophenyl groups leads to the highest increase in the luminescence quantum yields and decay times. This enhancement is particularly strong in the case of the Au corroles, where the phosphorescence quantum yield triples on going from Au[TPC] to Au[TPFPC]. An intriguing question concerns whether peripheral fluorination might have a similar positive effect on the luminescence properties of other porphyrin-type complexes such as true porphyrins, carbaporphyrins, hydroporphyrins, and dipyrrin derivatives. Time will tell.

## Data Availability

The data underlying this study are available in the published article and its Supporting Information.

## Supporting Information Available

Experimental methods, ^1^H and ^19^F NMR spectra, electrospray ionization mass spectra, optical spectra, and additional photophysical data (PDF)
