# Supplementary material for: Influence of Fluorinated Substituents on the Near-Infrared Phosphorescence of 5d Metallocorroles
Source: ACS Org Inorg Au. 2023 Jul 12;3(5):241–5. doi: 10.1021/acsorginorgau.3c00016 (PMC10557119; doi:10.1021/acsorginorgau.3c00016)
Supplement: Supplementary file 1 — gg3c00016_si_001.pdf [file gg3c00016_si_001.pdf]

Supporting Information

Influence of Fluorinated Substituents on the Near-Infrared Phosphorescence of 5d Metallocorroles

Krister Engedal Johannessen,[a] Martin Amund Langaas Johansen,[a] Rune F. Einrem,[a] Laura McCormick McPherson,[b] Abraham B. Alemayehu,*,[a] Sergey M. Borisov*,[c] and Abhik Ghosh*,[a]

[a]Department of Chemistry, UiT – The Arctic University of Norway, 9037 Tromsø, Norway;
[b]EPSRC National Crystallography Service, School of Chemistry, University of Southampton, Highfield, Southampton, SO17 1BJ, UK
[c]Institute of Analytical Chemistry and Food Chemistry, Graz University of Technology, Stremayrgasse 9, 8010 Graz, Austria

**Contents**	**Page**
A. Experimental section	S2
B. ^{1}H and ^{19}F NMR spectra	S6
C. Cyclic voltammograms	S13
D. ESI mass spectra	S15
E. Additional optical and photophysical measurements	S17

A. Experimental section

(a) Materials. Materials. Unless otherwise mentioned, all chemicals were obtained from Merck. Silica gel 60 (0.04-0.063 mm particle size, 230-400 mesh) was employed for flash chromatography. Silica gel 60 preparative thin-layer chromatographic plates (20 cm x 20 cm, 0.5 mm thick, Merck) were used for final purification of all complexes. Free-base corroles were synthesized according to previously reported procedures (*Org. Biomol. Chem.* **2003**, 1, 350-357; *J. Org. Chem.* **2005**, *71*, 3707-3717.).

(b) Instrumental methods. UV−visible spectra were recorded on an HP 8453 spectrophotometer. ^{1}H NMR spectra were recorded on a 400 MHz Bruker Avance III HD spectrometer equipped with a 5 mm BB/1H SmartProbe in $CDCl_3$ and referenced to residual $CHCl_3$ at 7.26 ppm. ^{19}F NMR spectra were acquired on the same spectrometer and referenced to hexafluorobenzene (C_6F_6, -164.9 ppm). High-resolution electrospray-ionization (HR-ESI) mass spectra were recorded from methanolic solution on an LTQ Orbitrap XL spectrometer.

Cyclic voltammetry was carried out at ambient temperature with a Gamry Reference 620 potentiostat equipped with a three-electrode system: A 3-mm glassy carbon disc working electrode, a platinum wire counterelectrode, and a saturated calomel reference electrode (SCE). Tetra(*n*-butyl)ammonium hexafluorophosphate was used as the supporting electrolyte, 0.1 M in dichloromethane. Anhydrous CH_2Cl_2 (Aldrich) was used as solvent. The electrolyte solution was purged with argon for at least 2 min prior to all measurements, which were carried out under an argon blanket. A 0.05 micrometer polishing alumina from ALS Japan was used and the glassy carbon working electrode was rubbed on top of a polishing pad. All potentials were referenced to the SCE.

The luminescence of the compounds was studied on a Fluorolog 3 fluorescence spectrometer from Horiba (Japan) equipped with a NIR-sensitive photomultiplier R2658 from Hamamatsu (Japan). Prior to measurements, toluene solutions of the complexes in sealable quartz cells (StarnaGmbH, Pfungstadt, Germany) were deoxygenated by bubbling high-purity nitrogen (99.99999%, Linde gas, Austria) for at least 15 min. Emission spectra were acquired upon excitation at the maximum of the Soret band. An OG 590 filter (Schott) was positioned in front of the emission channel to eliminate second-order grating artefacts. Excitation spectra were recorded on more diluted solutions (Soret absorbance ≤ 0.1) by monitoring in the maximum of the emission band. The luminescence quantum yields were determined relative to platinum(II) tetraphenyltetrabenzoporphyrin (Pt[TPTBP], $\Phi = 21\%$; see ref 37 of the main

paper). The ReO corroles were excited at 440 nm, whereas an excitation wavelength of 422 nm was used for OsN and Au corroles.

Luminescence decay times in solution were determined on the same spectrometer with the DeltaHub module (Horiba Scientific) controlling a SpectraLED-456 lamp (λ = 456 nm) and using DAS-6 analysis software for data analysis. 1129.0907

(c) Synthetic methods. All metalation procedures were adopted from our earlier work without modification (refs 5, 15 and 19 of the main paper). Analytical details for new compounds are as follows.

Au[T3,5-CF_3PC]. Light red crystalline solid, yield 24.1 mg (28.0%). UV-vis (CH_2Cl_2) λ_{max} [nm, ε x 10^{-4} ($M^{-1}cm^{-1}$)]: 421 (14.81)), 493 (0.060), 529 (1.20), 567 (3.26).^{1}H NMR (400 MHz, $CDCl_3$, 25 °C): (400 MHz, $CDCl_3$) δ 9.27 (d, 2H, J = 4.5 Hz, β-H), 8.95 (d, 2H, J = 4.9 Hz, β-H), 8.80 (d, 2H, J = 4.5 Hz, β-H), 8.74 (d, 6H, J = 5.7 Hz, β-H overlapping with 5,15-*o*-Ph), 8.66 (s, 2H, 10-*o*-Ph), 8.34 (s, 3H, 5,10,15-*p*-Ph); ^{19}F NMR -65.58 (s, 18F, 5,10,15(3,5-CF3). HRMS (ESI-TOF) m/z: $[M + H]^+$ Calcd for $C_{43}H_{18}F_{18}N_4Au$ 1129.0904; found 1129.0907.

Re[T3,5-CF_3PC](O). Dark red crystalline solid, yield 26.8 mg (22.4%). UV-vis (CH_2Cl_2) λ_{max} [nm, ε x 10^{-4} ($M^{-1}cm^{-1}$)]: 437 (10.99), 553 (1.85), 584 (2.10). ^{1}H NMR (400 MHz, $CDCl_3$, 25 °C) δ 9.79 (d, 2H, J = 4.5 Hz, β-H), 9.32 (d, 2H, J = 4.5 Hz, β-H), 9.28 (d, 2H, J = 4.9 Hz, β-H), 9.10 (d, 4H, J = 4.9 Hz, β-H overlapping with 5,15-*o*1-Ph), 9.04 (s, 1H, 10-*o*1-Ph), 8.55 (s, 2H, 5,15-*o*2-Ph), 8.39 (s, 4H, 10-*o*2-Ph overlapping with 5,10,15-*p*-Ph); ^{19}F NMR -65.72 (s, 18F, 5,10,15(3,5-CF_3). HRMS (ESI-TOF) m/z: $[M + H]^+$ Calcd for $C_{43}H_{18}F_{18}N_4ORe$ 1135.0747; found 1135.0748.

Re[TPFPC](O). Dark red crystalline solid, yield 34 mg (50.89%). UV-vis (CH_2Cl_2) λ_{max} [nm, ε x 10^{-4} ($M^{-1}cm^{-1}$)]: 326 (1.52), 434 (8.71), 550 (1.18), 583 (1.31). ^{1}H NMR (400 MHz, $CDCl_3$, 25 °C) δ 9.75 (d, J = 4.5 Hz, 2H, β-H), 9.32 (d, J = 4.6 Hz, 2H, β-H), 9.25 (d, J = 5.0 Hz, 2H, β-H), 9.14 (d, J = 4.9 Hz, 2H, β-H); ^{19}F NMR -136.03 (dddd, J = 43.4, 23.8, 8.9., 3.7 Hz, 3F, 5,10,15-*o*1-PF), -137.29-137.93 (m, 3F, 5,10,15-*o*2-PF), -151.99 (dt, J = 27.7, 21.1 Hz, 3F, 5,10,15-*p*-PF), -160.89 (tdd, J = 21.5, 12.5, 8.5 Hz, 3F, 5,10,15-*m*1-PF), -161.17 (dddd, J = 44.9, 23.7, 20.9, 8.6Hz, 3F, 5,10,15-*m*2-PF. HRMS (ESI-TOF) m/z: $[M + H]^+$ Calcd for $C_{37}H_9F_{15}N_4ORe$ 996.0014; found 996.0012.

Os[T3,5-CF_3PC](N). Red crystalline solid, yield 36.9 mg (35.1%). UV-vis (CH_2Cl_2) λ_{max} [nm, ε x 10^{-4} ($M^{-1}cm^{-1}$)]: 440 (11.52), 553 (1.51), 585 (2.22). ^{1}H NMR (400 MHz, $CDCl_3$)

δ 9.70 (d, 2H, J = 4.5 Hz, β-H), 9.27 (d, 2H, J = 5.0 Hz, β-H), 9.21 (d, 2H, J = 4.5 Hz, β-H), 9.08 (s, 2H, 5,15-*o*1-Ph), 9.05 (d, 2H, J = 5.0 Hz β-H), 9.01 (s, 1H, 10-*o*1-Ph), 8.58 (s, 2H, 5,15-*o*2-Ph), 8.46 (s, 1H, 10-*o*2-Ph), 8.39 (s, 3H, 5,10,15-*p*-Ph); ^{19}F NMR -65.55 (s, 18F, 5,10,15(3,5-CF_3). HRMS (ESI-TOF) m/z: $[M + H]^+$ Calcd for $C_{43}H_{18}F_{18}N_5Os$ 1138.0887; found 1138.0887.

X-ray structure determinations. X-ray quality crystal was grown by slow diffusion of methanol into concentrated solution of Re[T3,5-CF_3PC](O) in dichloromethane and X-ray data were collected at the National Crystallography Service at the University of Southampton. The crystal was coated in protective perfluoroether oil before being mounted on a MiTeGen loop and transferred to the goniometer head of a Rigaku 007HF diffractometer equipped with Varimax confocal mirrors, an AFC11 goniometer, and a HyPix 6000 detector. The sample was held at a temperature of 100(2) K with an Oxford Cryosystems CryostreamPlus device. Crystallographic data were measured using profile data from ω scans using Cu Kα radiation. The total number of runs and images was based on strategy calculation from the Rigaku's CrysAlisPro program. The structure was solved with intrinsic phasing methods (with SHELXT as described in: *Acta Cryst.* **2015**, *A71*, 3-8) and refined by full matrix least squares on F^2 (with SHELXL-2018 as described in: *Acta Cryst.* **2015**, *C71*, 3-8) using the ShelXle GUI. Hydrogen atoms were included at their geometrically estimated positions. One CF_3 group was found to be disordered, and the two sites refined were with complementary occupancies. The C-F and F-F bonds were restrained to be equal across the two sites, and corresponding pairs of atoms (e.g. F17 in the two sites) were constrained to have equal anisotropic thermal displacement parameters. The two C(pyrrole)-C(CF_3) bond lengths were restrained to have equal length.

Table S1. Crystal and refinement data for Re[T3,5-CF_3PC](O).

Empirical formula	$C_{43}H_{17}F_{18}N_4ORe$
Formula weight	1133.80
Temperature	100(2) K
Wavelength	1.54184 Å
Crystal system	Triclinic
Space group	P
Unit cell dimensions	a = 8.2583(2) Å □= 73.4740(10)°<br>b = 14.6249(3) Å □= 89.726(2)°<br>c = 17.9347(2) Å □ = 85.316(2)°.
Volume	2069.30(7) Å3
Z	2
Density (calculated)	1.820 Mg/m3
Absorption coefficient	6.866 mm-1
F(000)	1096
Crystal size	0.110 x 0.040 x 0.020 mm3
Theta range for data collection	2.570 to 70.662°
Index ranges	$-10 \leq h \leq 10$, $-17 \leq k \leq 17$, $-21 \leq l \leq 20$
Reflections collected	88606
Independent reflections	7781 [R(int) = 0.0575]
Completeness to theta = 67.684°	99.5 %
Absorption correction	Analytical
Max. and min. transmission	0.875 and 0.661
Refinement method	Full-matrix least-squares on F2
Data / restraints / parameters	7781 / 7 / 617
Goodness-of-fit on F2	1.054
Final R indices [I > 2sigma(I)]	R1 = 0.0354, wR2 = 0.0943
R indices (all data)	R1 = 0.0378, wR2 = 0.0957
Extinction coefficient	n/a
Largest diff. peak and hole	1.157 and -1.549 e.Å-3

B. ^{1}H and ^{19}F NMR spectra

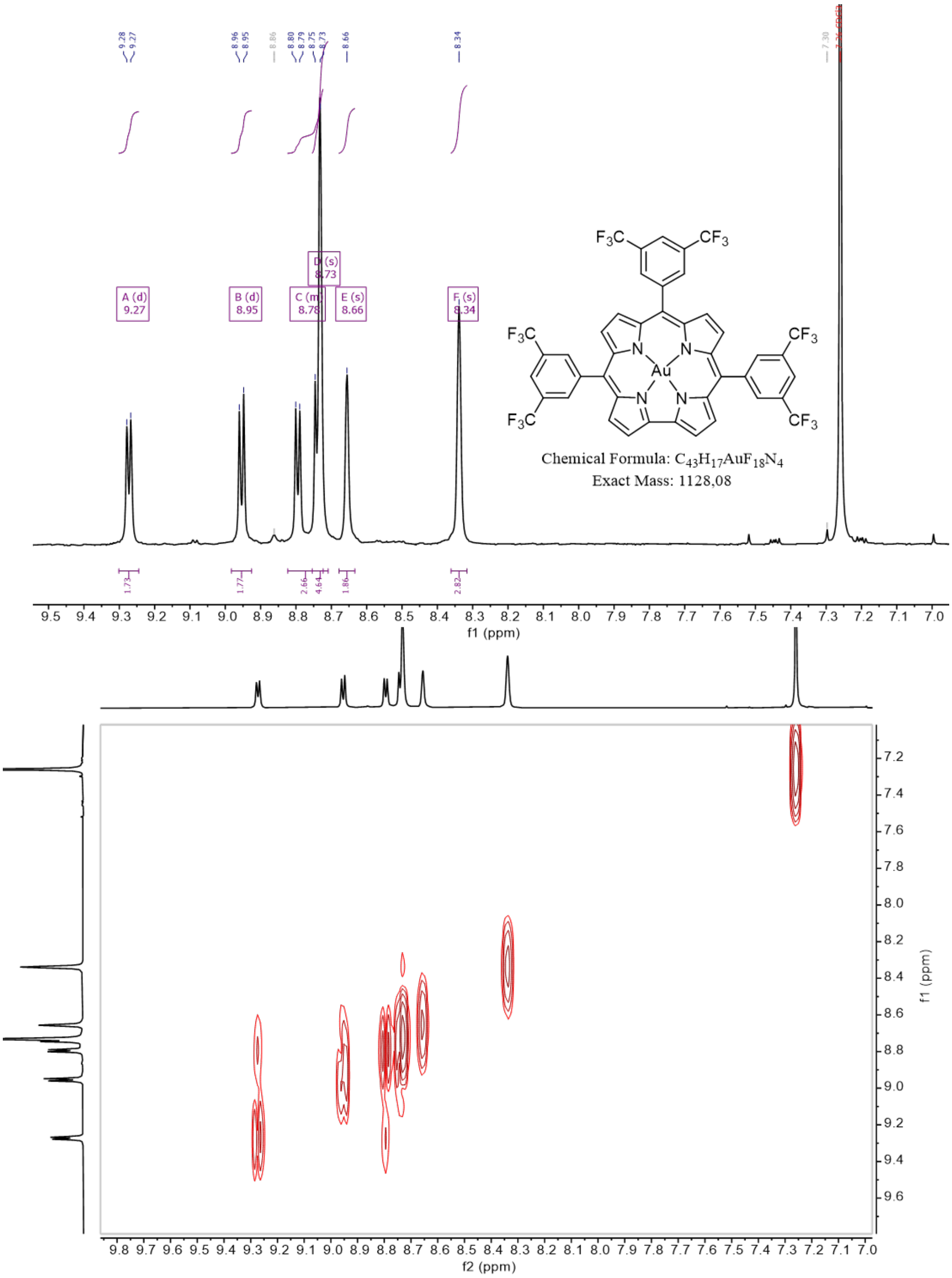

Figure S1. ^{1}H NMR spectrum and ^{1}H-^{1}H COSY of Au[T3,5-CF_3PC] in $CDCl_3$ at room temperature on a 400-MHz spectrometer.

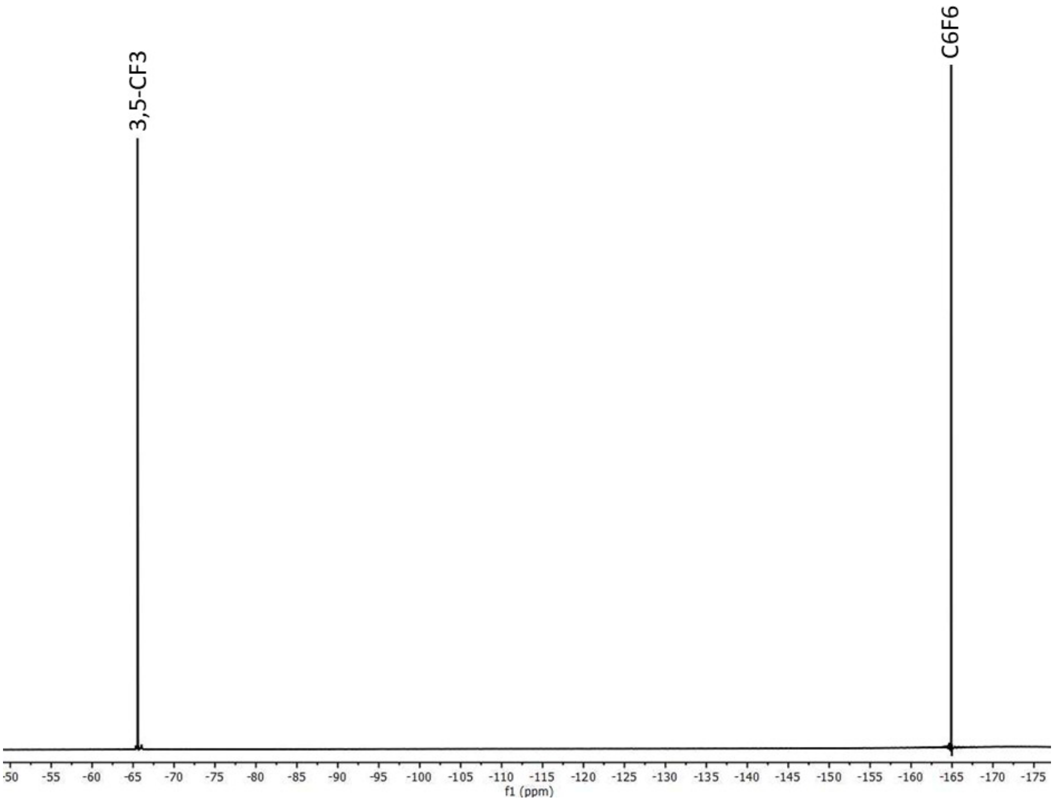

Figure S2. ^{19}F NMR spectrum of Au[T3,5-CF_3PC].

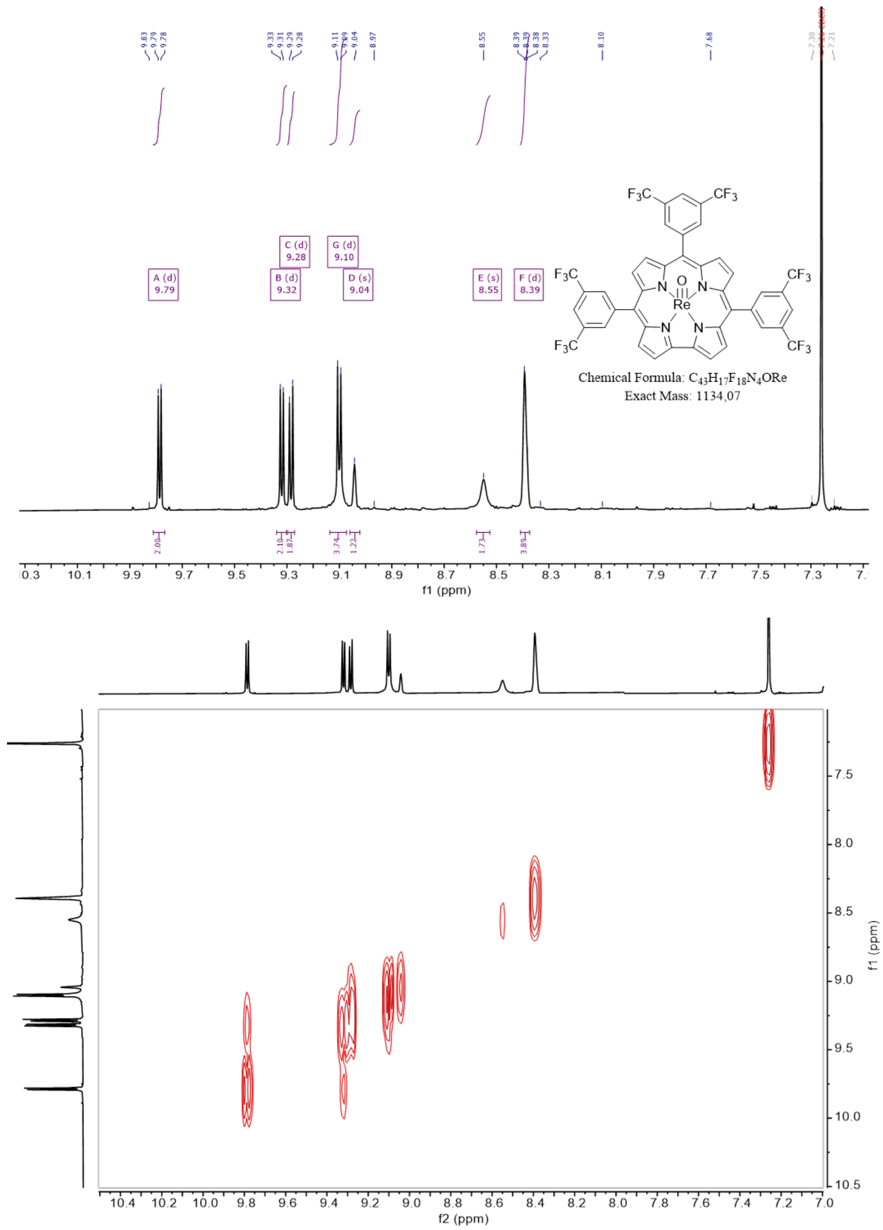

Figure S3. ^{1}H NMR spectrum and ^{1}H-^{1}H COSY of Re[T3,5-CF_3PC](O) in $CDCl_3$ at room temperature on a 400-MHz spectrometer.

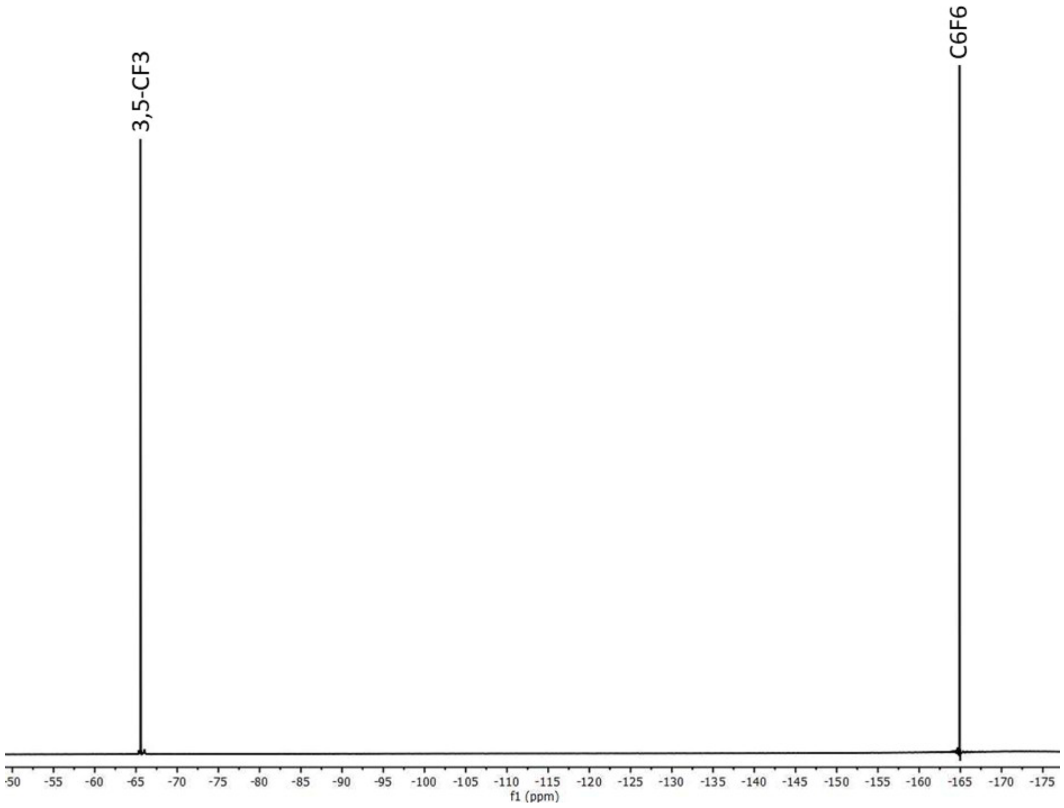

Figure S4. ^{19}F NMR spectrum of Re[T3,5-CF_3PC](O).

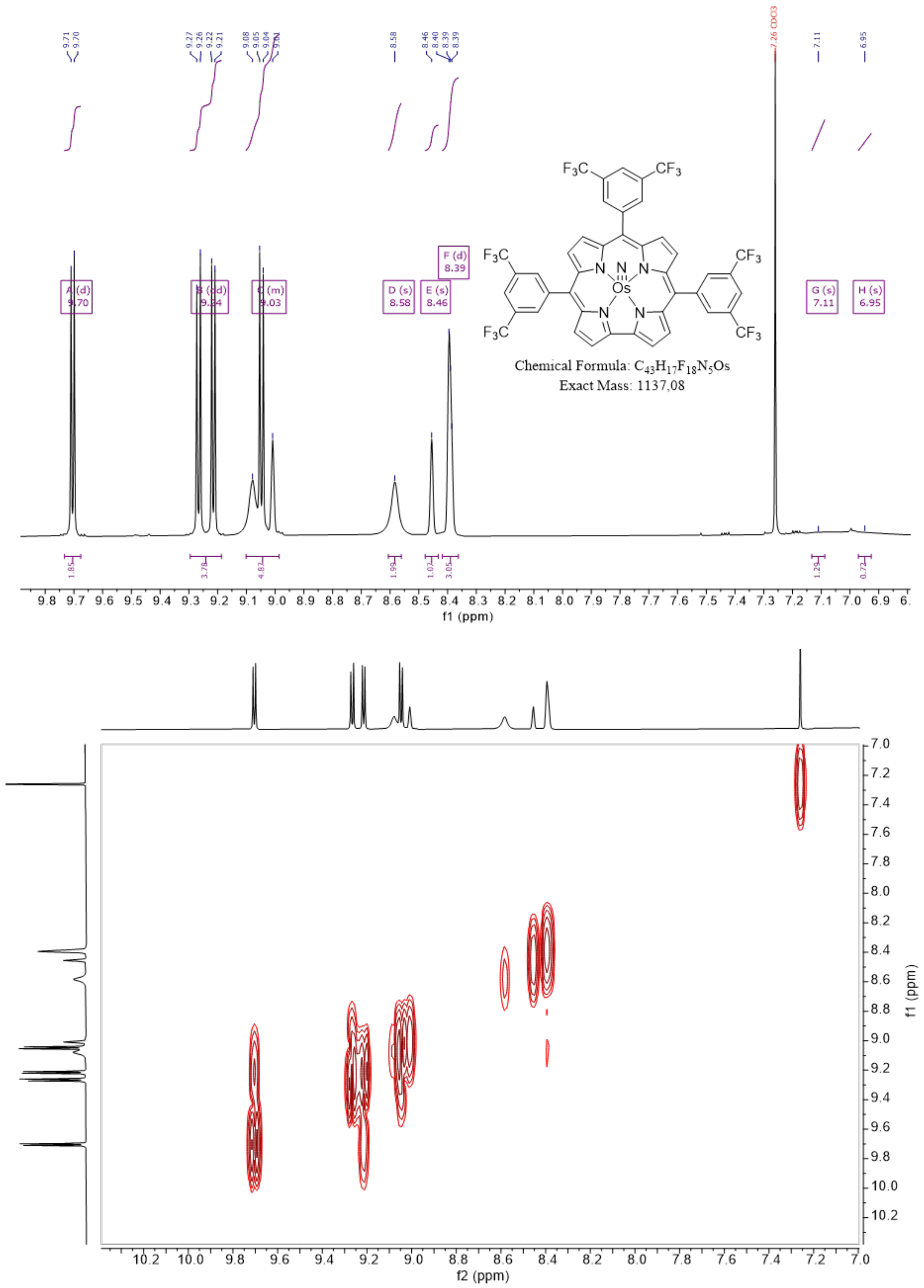

Figure S5. ^{1}H NMR spectrum and ^{1}H-^{1}H COSY spectra of Os[T(3,5-CF_3)PC](N) in $CDCl_3$ at room temperature on a 400-MHz spectrometer.

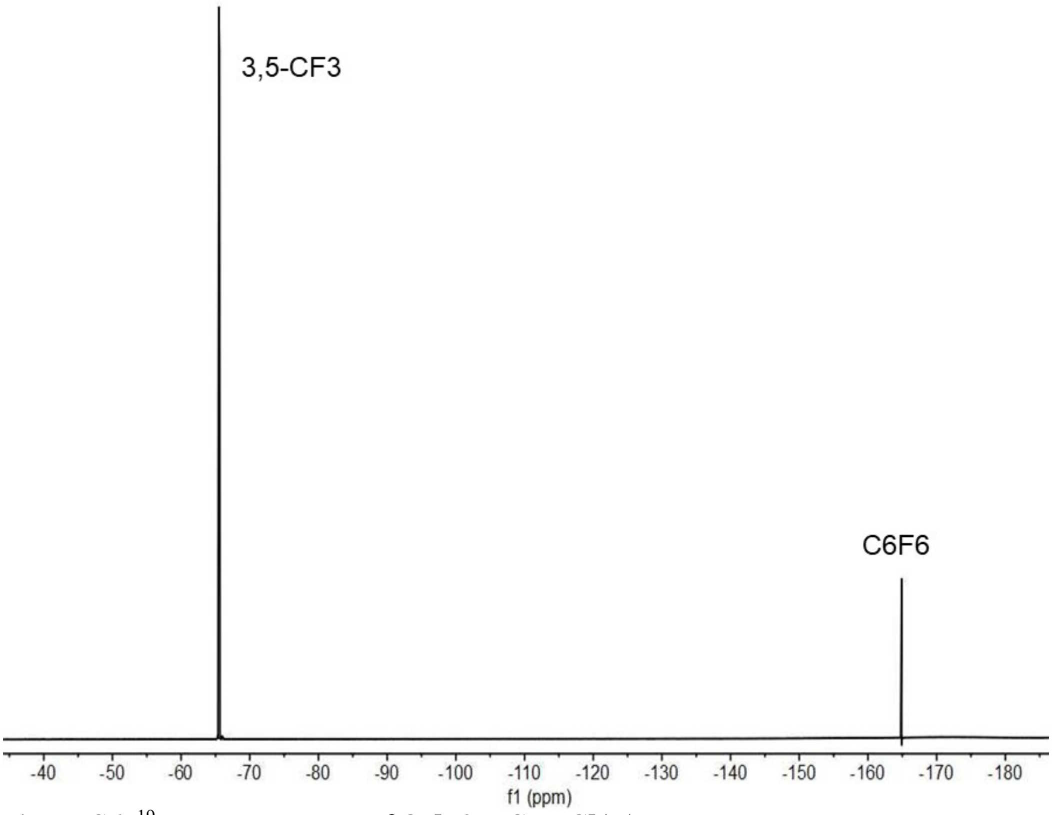

Figure S6. ^{19}F NMR spectrum of Os[T3,5-CF_3PC](N).

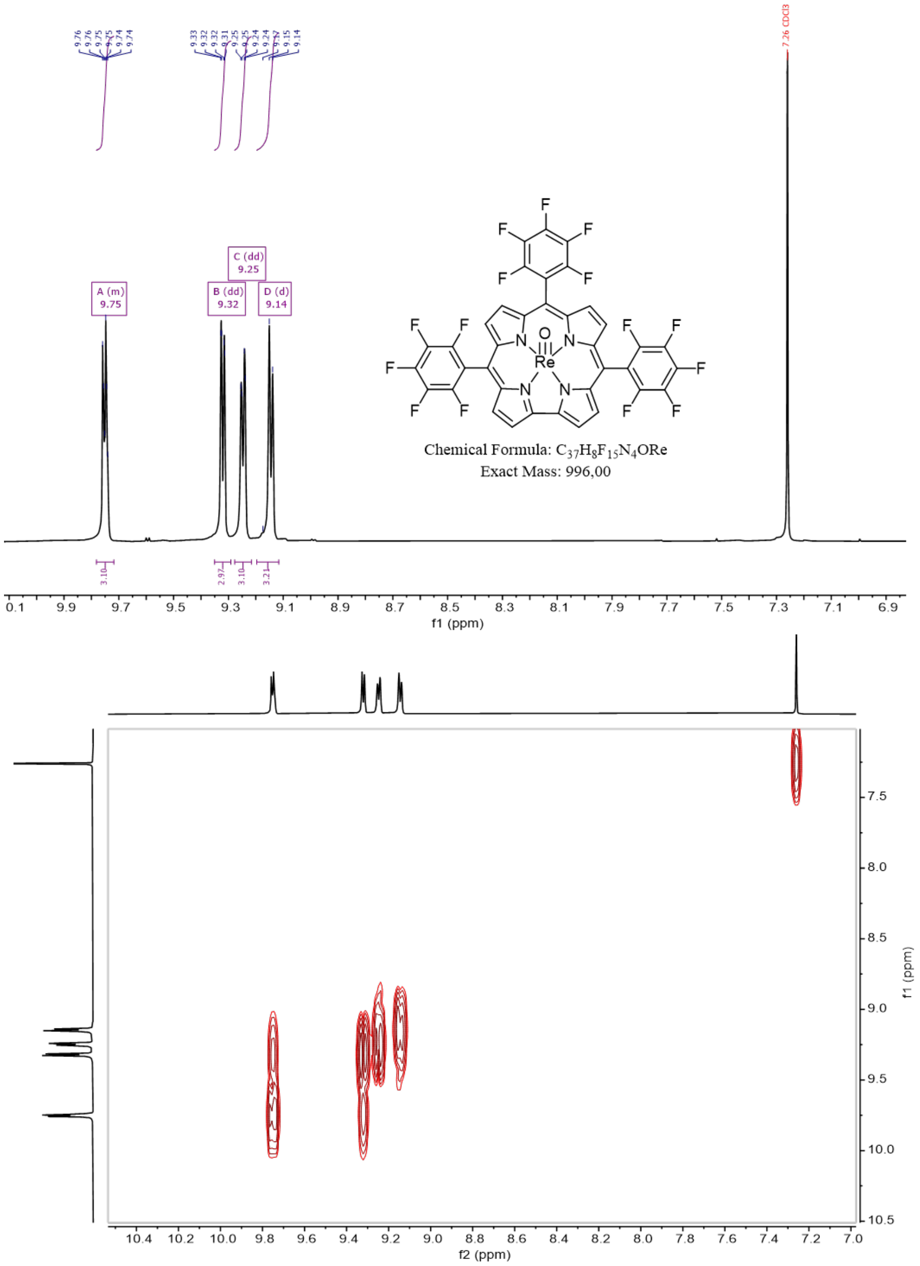

Figure S7. ^{1}H NMR spectrum and ^{1}H-^{1}H COSY of Re[TPFPC](O) in $CDCl_3$ at room temperature on a 400-MHz spectrometer.

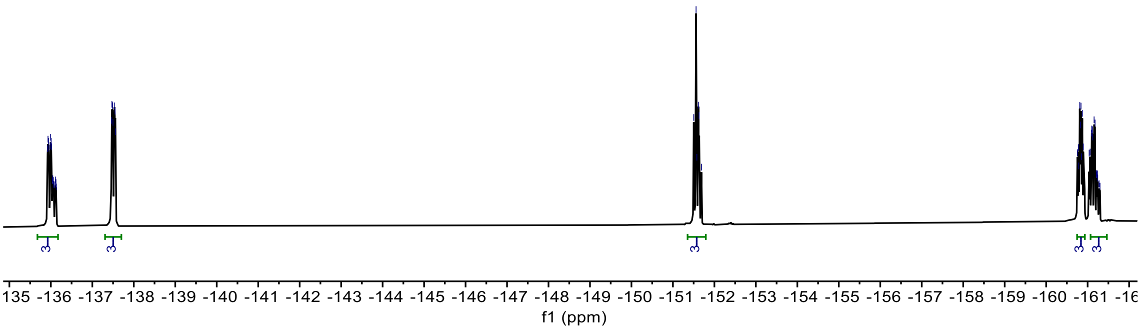

Figure S8. ^{19}F NMR spectrum of Re[TPFPC](O).

C. Cyclic voltammograms

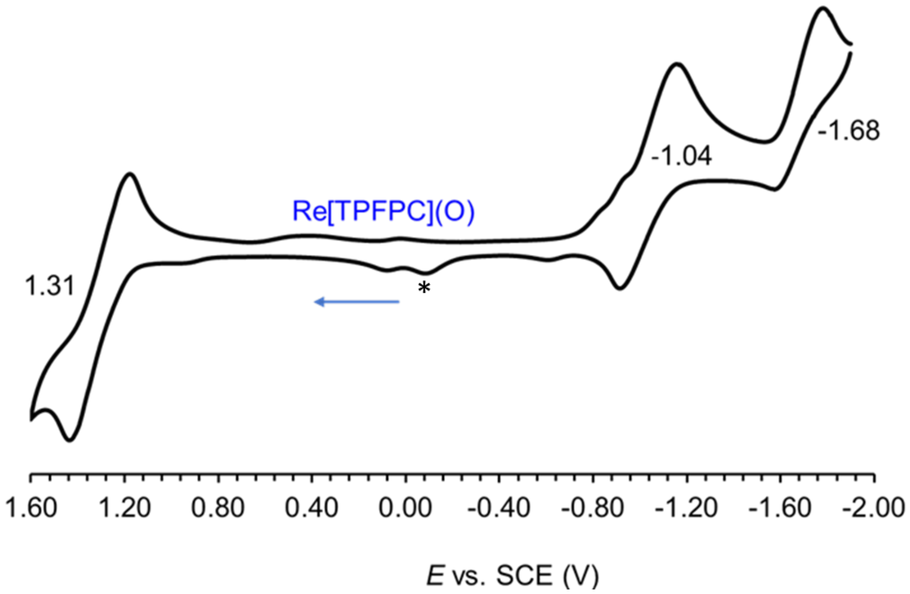

Figure S9. Cyclic voltammogram of Re[TPFPC](O) in CH_2Cl_2 containing 0.1 M TBAP; scan rate 100 mV·s^{-1} at room temperature. The starred feature in the CV is due to irreversibility of the second reduction. Experimental details are given under Instrumental Methods (see p S2).

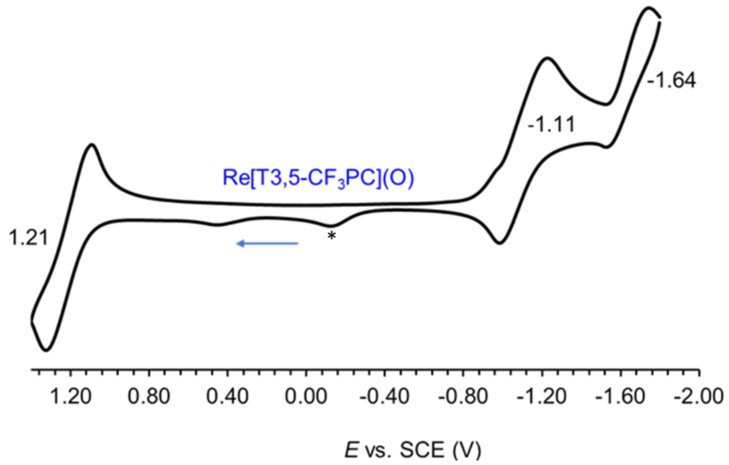

Figure S10. Cyclic voltammogram of Re[T3,5-CF_3PC](O) in CH_2Cl_2 containing 0.1 M TBAP; scan rate 100 mV·s^{-1} at room temperature. The starred feature in the CV is due to irreversibility of the second reduction. Experimental details are given under Instrumental Methods (see p S2).

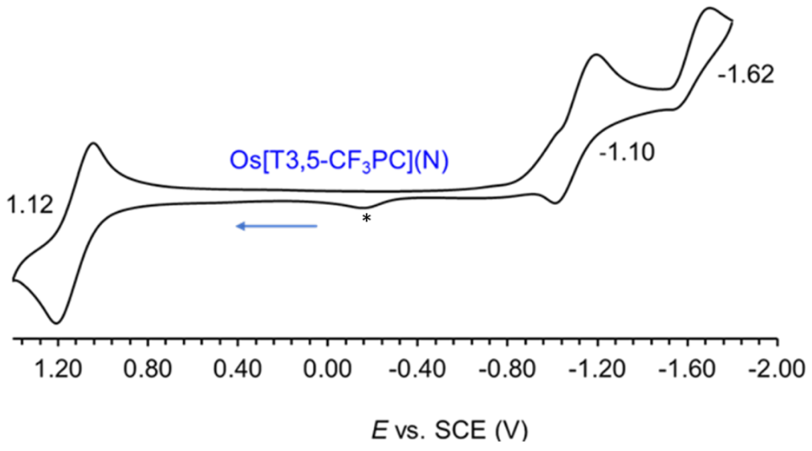

Figure S11. Cyclic voltammogram of Os[T3,5-CF$_3$PC](N) in CH_2Cl_2 containing 0.1 M TBAP; scan rate 100 mV·s $^{-1}$ at room temperature. The starred feature in the CV is due to irreversibility of the second reduction. Experimental details are given under Instrumental Methods (see p S2).

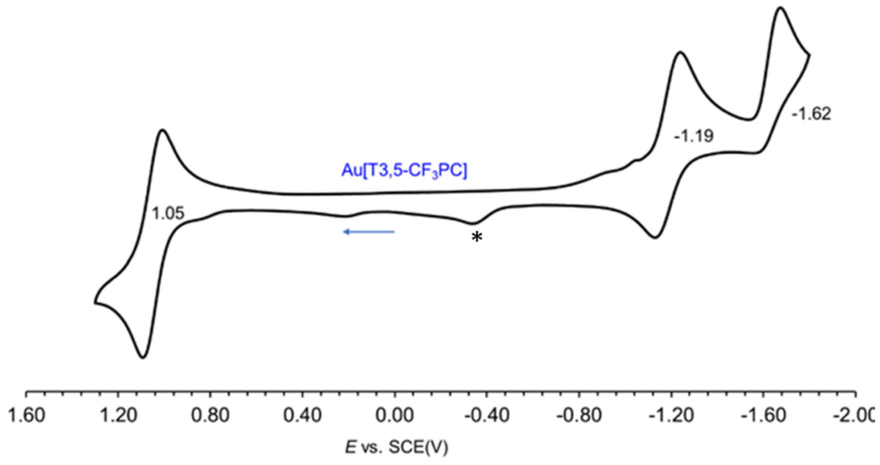

Figure S12. Cyclic voltammogram of Au[T3,5-CF$_3$PC] in CH_2Cl_2 containing 0.1 M TBAP; scan rate 100 mV·s $^{-1}$ at room temperature. A 3-mm glassy carbon disc working electrode, a platinum wire counterelectrode, and a saturated calomel reference electrode, (SCE) were employed for the experiment. The * in the CV is due to the irreversibility of the second reduction.

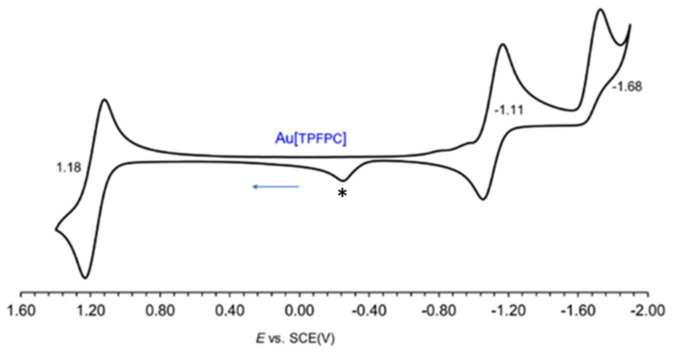

Figure S13. Cyclic voltammogram of Au[TPFPC] in CH_2Cl_2 containing 0.1 M TBAP; scan rate 100 mV·s $^{-1}$ at room temperature. The starred feature in the CV is due to irreversibility of the second reduction. Experimental details are given under Instrumental Methods (see p S2).

D. ESI mass spectra

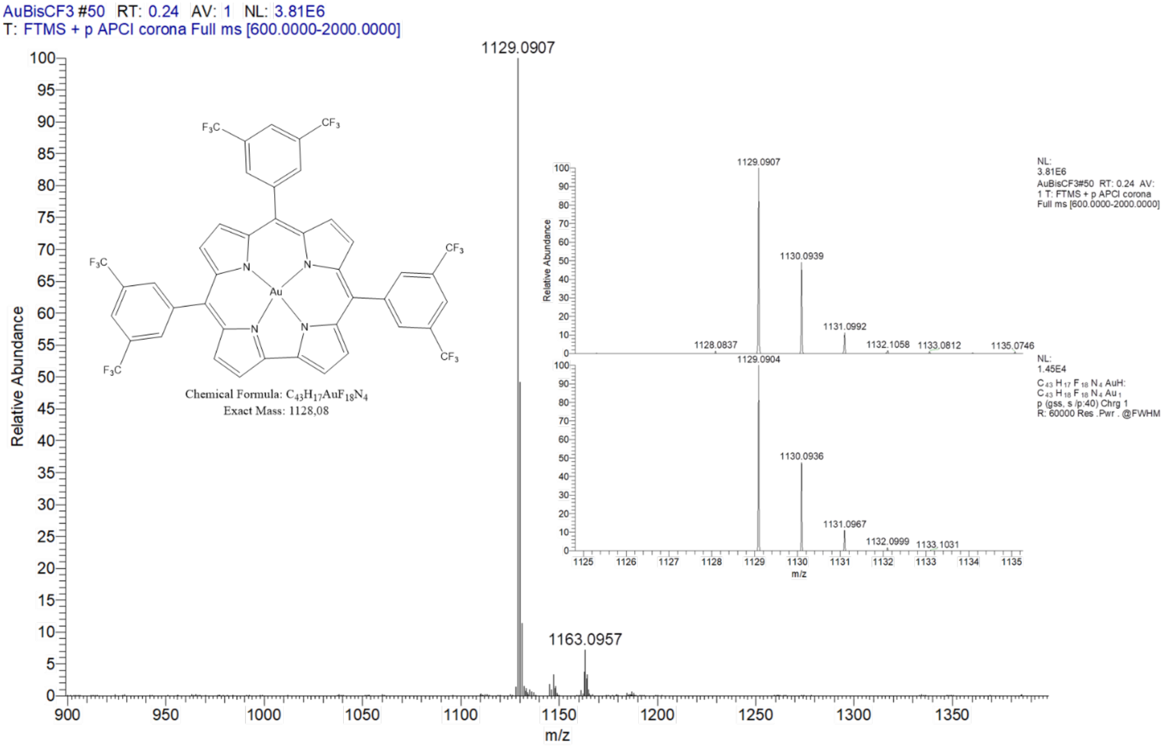

Figure S14. ESI-MS of Au[T3,5-CF_3PC]. Detail of $[M+H]^+$ (above), with simulation (below).

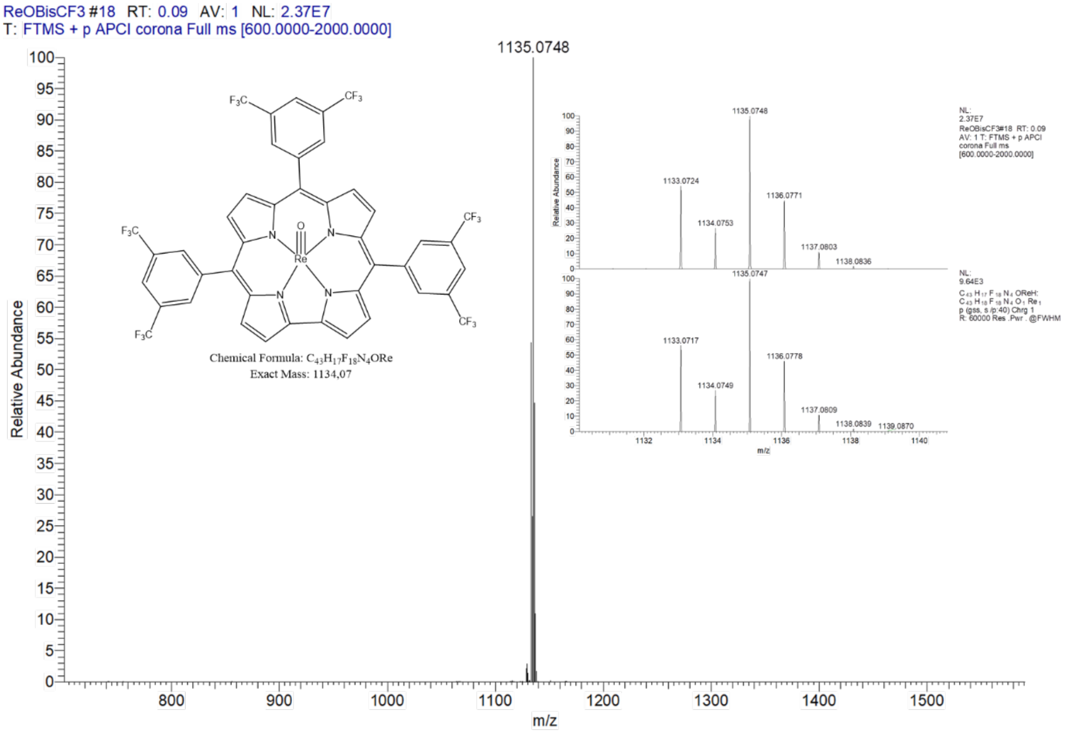

Figure S15. ESI-MS of Re[T(3,5-CF_3P)C](O). Detail of $[M+H]^+$ (above), with simulation (below).

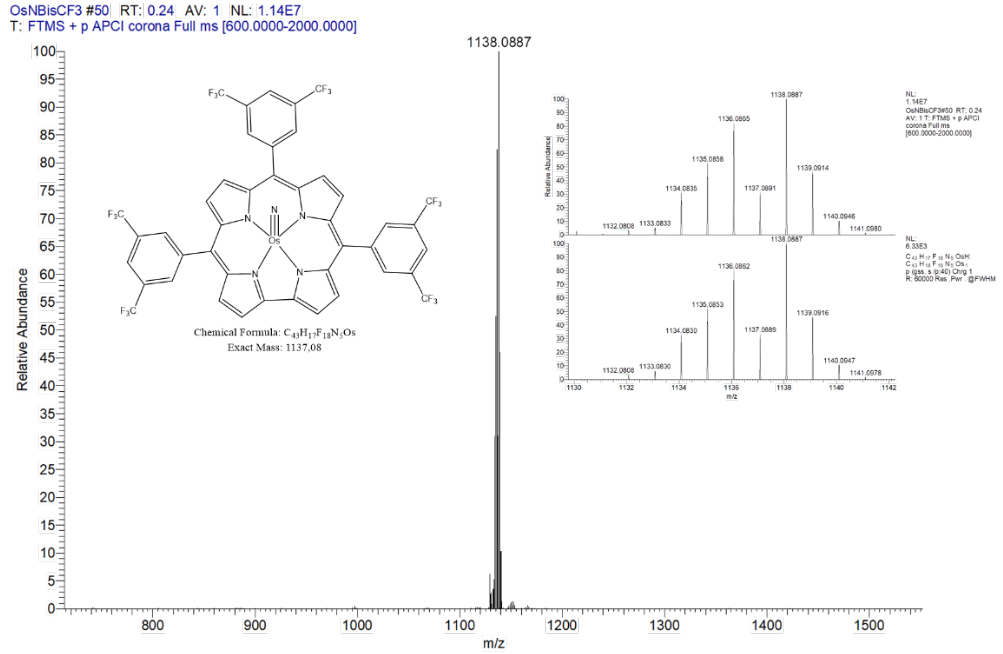

Figure S16. ESI-MS of Os[T(3,5-CF_3P)C](N). Detail of $[M+H]^+$ (above), with simulation (below).

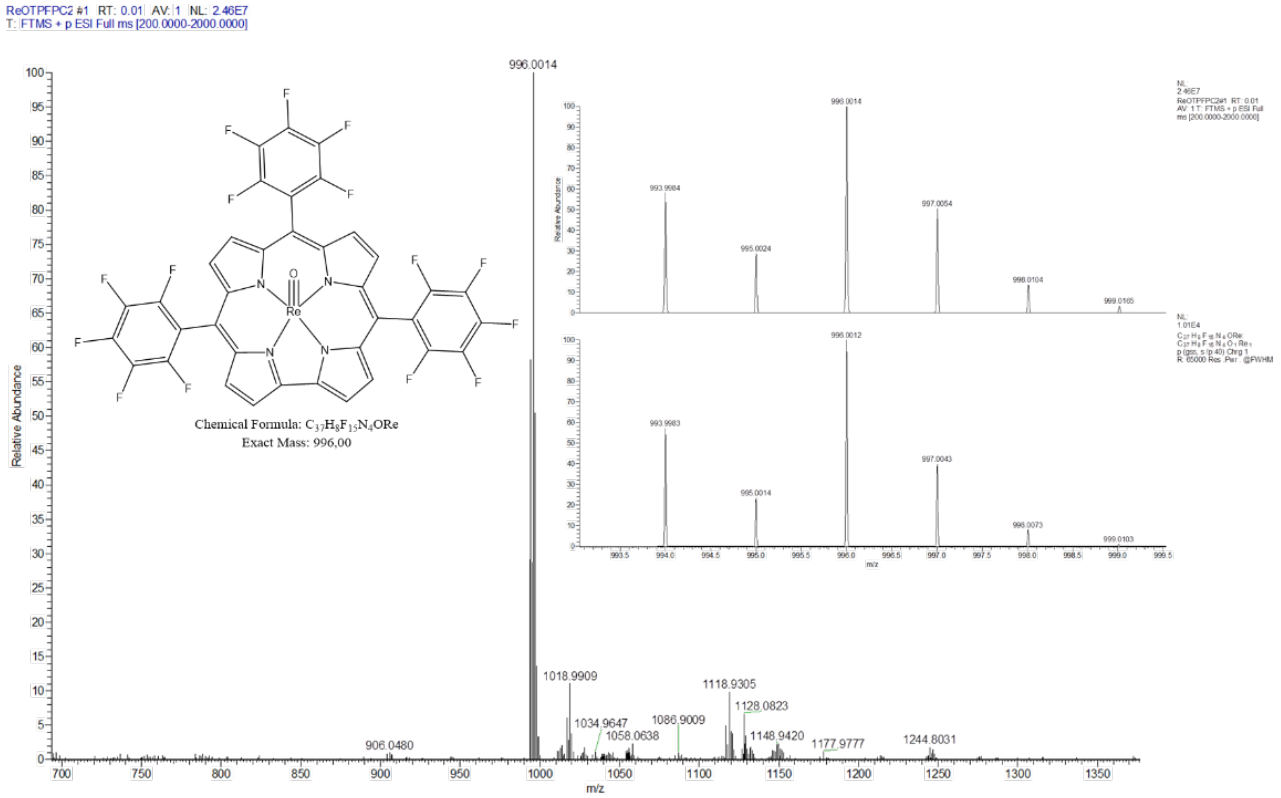

Figure S17. ESI-MS of Re[TPFPC](O). Detail of $[M+H]^+$ (above), with simulation (below).

E. Additional optical and photophysical measurements

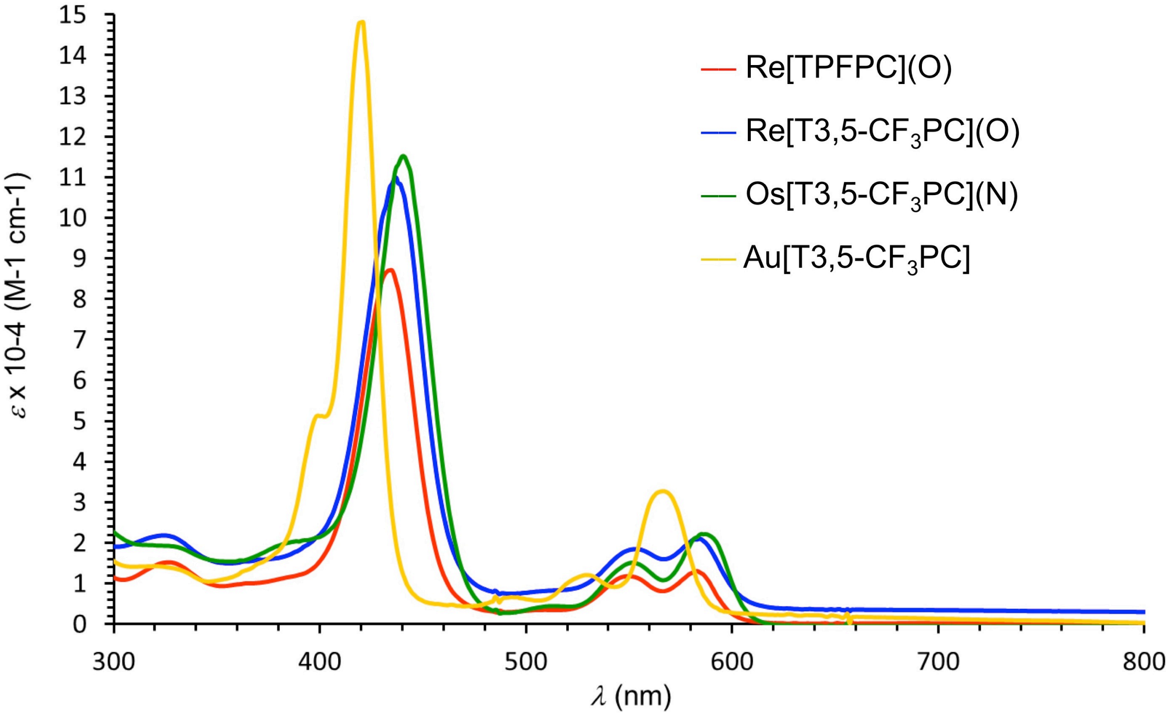

Figure S18. UV-vis spectra of Re[TPFPC](O), Re[T3,5-CF_3PC](O), Os[T3,5-CF_3PC](N) and Au[T3,5-CF_3PC] in dichloromethane.

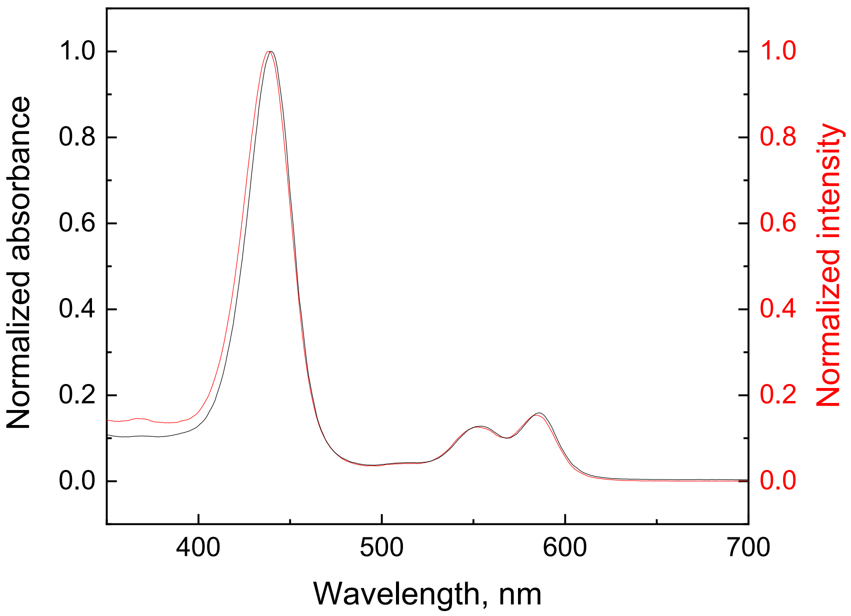

Figure S19. Normalized absorption and excitation spectra (λ_{em} = 760 nm) of Re[T3,5-CF_3PC](O) in toluene. The excitation spectrum was acquired under anoxic conditions.

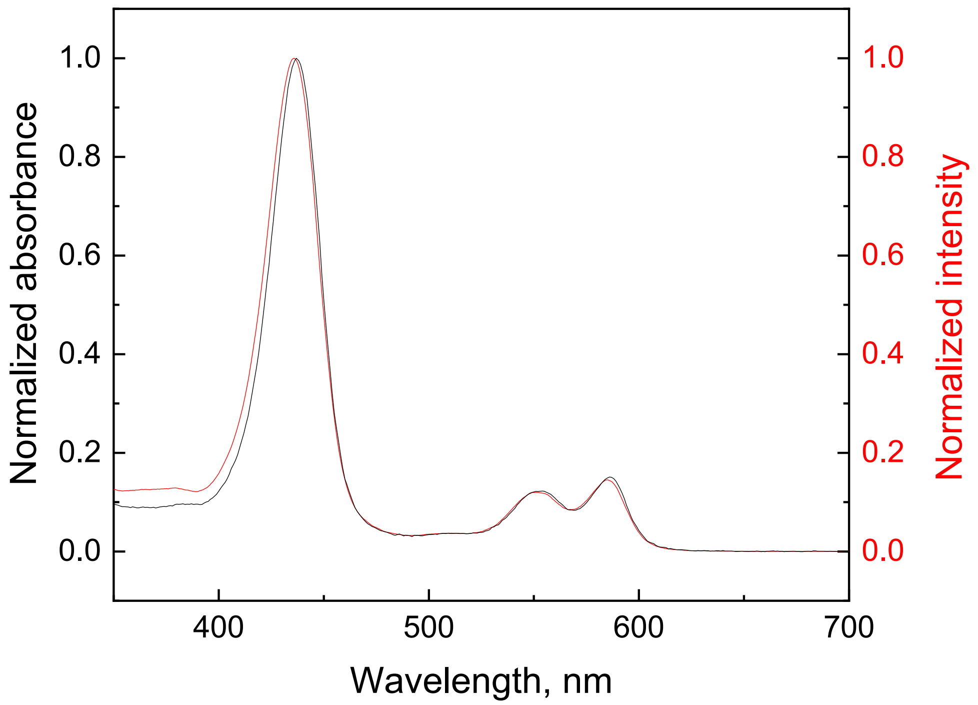

Figure S20. Normalized absorption and excitation spectra (λ_{em} = 753 nm) of Re[TPFPC](O) in toluene. The excitation spectrum was acquired under anoxic conditions.

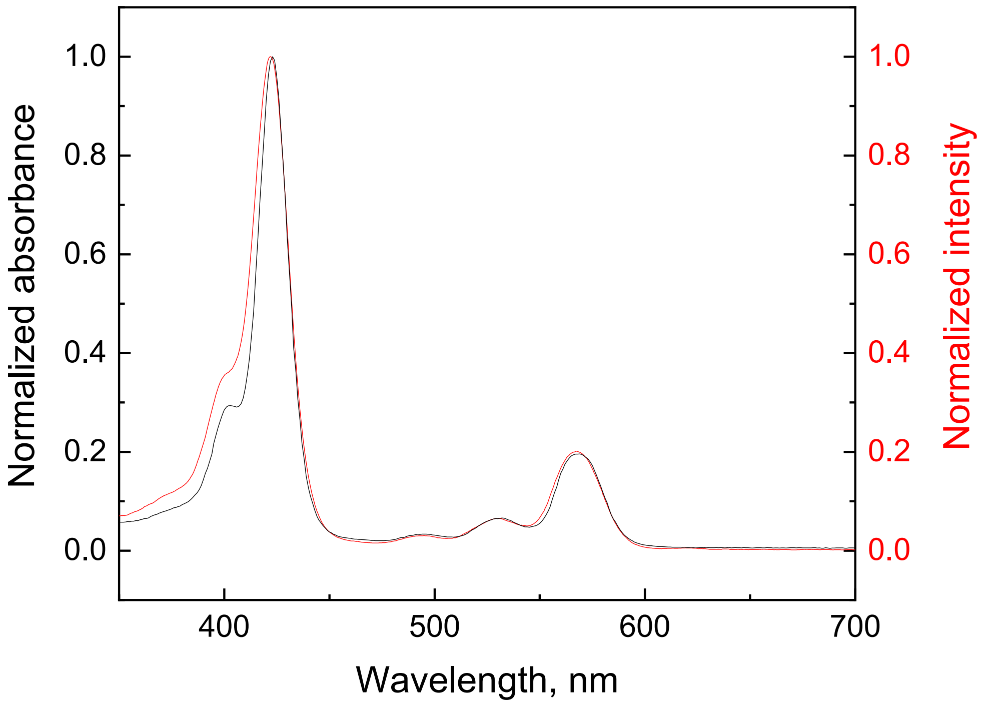

Figure S21. Normalized absorption and excitation spectra (λ_{em} = 777 nm) of Au[T3,5-CF_3PC] in toluene. The excitation spectrum was acquired under anoxic conditions.

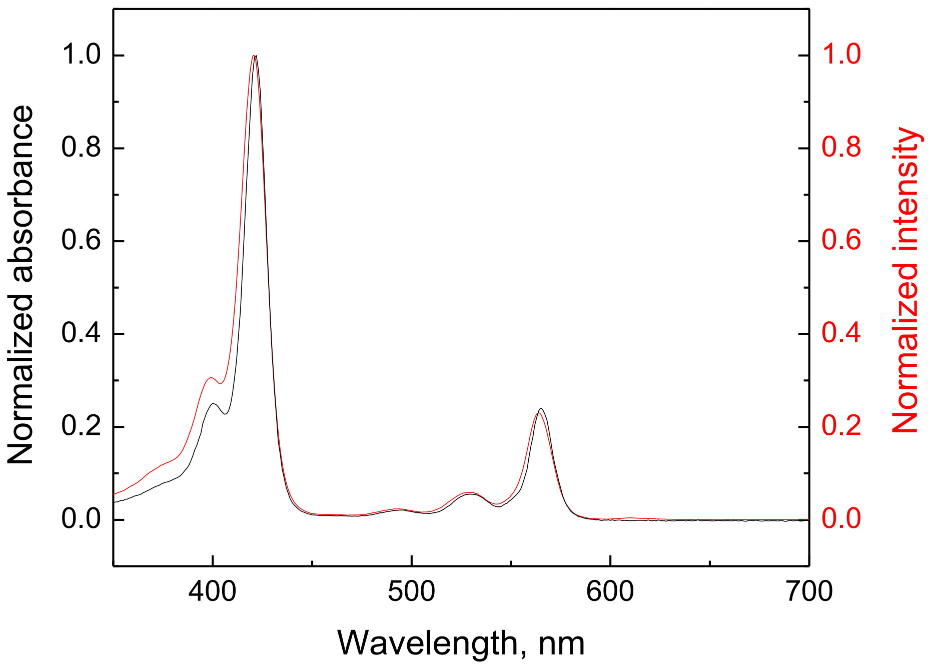

Figure S22. Normalized absorption and excitation spectra (λ_{em} = 751 nm) of Au[TPFPC] in toluene. The excitation spectrum was acquired under anoxic conditions.

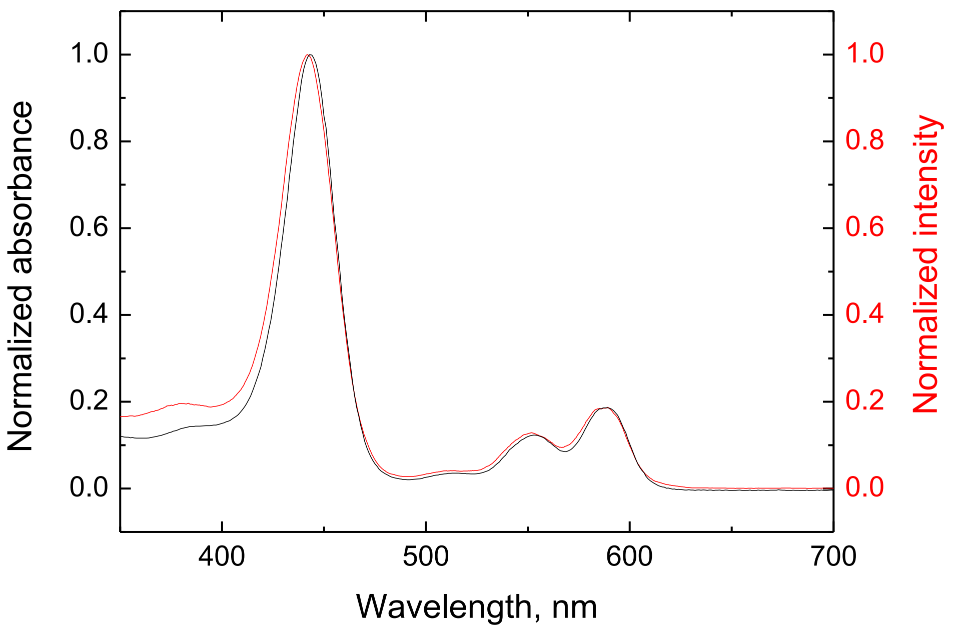

Figure S23. Normalized absorption and excitation spectra (λ_{em} = 751 nm) of Os[T3,5-CF_3PC](N) in toluene. The excitation spectrum was acquired under anoxic conditions.

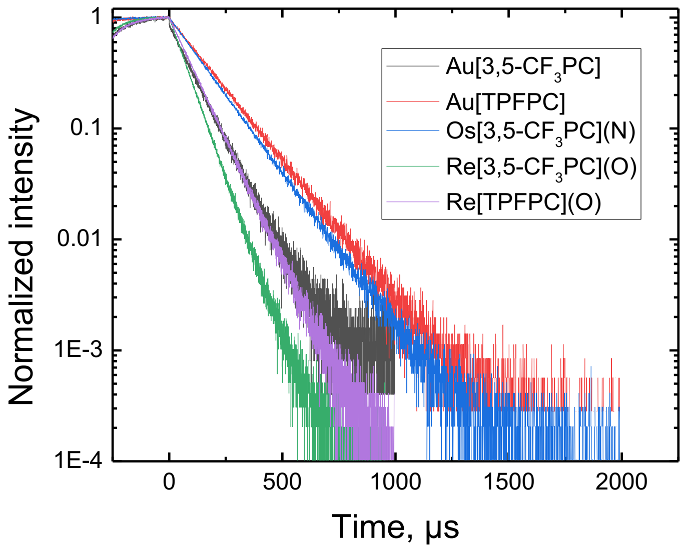

Figure S24. Luminescence decays (anoxic toluene, 23 °C) of new Au, OsN and ReO complexes with fluorinated substituents.
